# Hepatitis C Virus Antiviral Drug Resistance and Salvage Therapy Outcomes Across Australia

**DOI:** 10.1093/ofid/ofae155

**Published:** 2024-03-18

**Authors:** Dao Sen Wang, Amy Phu, Kristen McKee, Simone I Strasser, Sinead Sheils, Martin Weltman, Sue Sellar, Joshua S Davis, Mel Young, Alicia Braund, Geoffrey C Farrell, Anne Blunn, Damian Harding, Lucy Ralton, Kate Muller, Scott A Davison, David Shaw, Marnie Wood, Krispin Hajkowicz, Richard Skolen, Jane Davies, Jaclyn Tate-Baker, Adam Doyle, Rhoda Tuma, Simon Hazeldine, Wendy Lam, Natalie Edmiston, Krista Zohrab, William Pratt, Belinda Watson, Amany Zekry, Carlie Stephens, Paul J Clark, Melany Day, Gordon Park, Hami Kim, Mark Wilson, Bruce McGarity, Natalie Menzies, Darren Russell, Thao Lam, Peter Boyd, Jen Kok, Jacob George, Mark W Douglas

**Affiliations:** Storr Liver Centre, The Westmead Institute for Medical Research, The University of Sydney and Westmead Hospital, Sydney, NSW, Australia; Storr Liver Centre, The Westmead Institute for Medical Research, The University of Sydney and Westmead Hospital, Sydney, NSW, Australia; Storr Liver Centre, The Westmead Institute for Medical Research, The University of Sydney and Westmead Hospital, Sydney, NSW, Australia; AW Morrow Gastroenterology and Liver Centre, The University of Sydney and Royal Prince Alfred Hospital, Camperdown, NSW, Australia; AW Morrow Gastroenterology and Liver Centre, The University of Sydney and Royal Prince Alfred Hospital, Camperdown, NSW, Australia; Department of Gastroenterology and Hepatology, Nepean Hospital, Kingswood, NSW, Australia; Department of Gastroenterology and Hepatology, Nepean Hospital, Kingswood, NSW, Australia; Department of Infectious Diseases, University of Newcastle and John Hunter Hospital, Newcastle, NSW, Australia; Department of Infectious Diseases, University of Newcastle and John Hunter Hospital, Newcastle, NSW, Australia; Department of Gastroenterology and Hepatology, Gold Coast University Hospital, Southport, QLD, Australia; Department of Gastroenterology and Hepatology, Australian National University and The Canberra Hospital, Canberra, ACT, Australia; Department of Gastroenterology and Hepatology, Australian National University and The Canberra Hospital, Canberra, ACT, Australia; Department of Gastroenterology and Hepatology, Lyell McEwin Hospital, Elizabeth Vale, SA, Australia; Department of Gastroenterology and Hepatology, Lyell McEwin Hospital, Elizabeth Vale, SA, Australia; Department of Gastroenterology and Hepatology, Flinders Medical Centreand Flinders University, Adelaide, SA, Australia; Department of Gastroenterology and Hepatology, University of New South Wales and Liverpool Hospital, Liverpool, NSW, Australia; Department of Infectious Diseases, Royal Adelaide Hospital, Adelaide, SA, Australia; Infectious Diseases Unit, Royal Brisbane and Women's Hospital, Brisbane, QLD, Australia; Infectious Diseases Unit, Royal Brisbane and Women's Hospital, Brisbane, QLD, Australia; Infectious Diseases Unit, Royal Brisbane and Women's Hospital, Brisbane, QLD, Australia; Menzies School of Health Research and Royal Darwin Hospital, Darwin, NT, Australia; Menzies School of Health Research and Royal Darwin Hospital, Darwin, NT, Australia; Department of Gastroenterology and Hepatology, Royal Perth Hospital, Perth, WA, Australia; Department of Gastroenterology and Hepatology, Royal Perth Hospital, Perth, WA, Australia; Department of Gastroenterology and Hepatology, Fiona Stanley Hospital, Murdoch, WA, Australia; Department of Gastroenterology and Hepatology, Fiona Stanley Hospital, Murdoch, WA, Australia; Department of Gastroenterology and Hepatology, School of Medicine, Western Sydney University, Sydney, NSW, Australia; Department of Gastroenterology and Hepatology, School of Medicine, Western Sydney University, Sydney, NSW, Australia; Department of Medicine, Shoalhaven Hospital, Nowra, NSW, Australia; Department of Medicine, Shoalhaven Hospital, Nowra, NSW, Australia; Department of Gastroenterology and Hepatology, St George Hospital, Kogarah, NSW, Australia; Department of Gastroenterology and Hepatology, St George Hospital, Kogarah, NSW, Australia; Rockhampton Blood Borne Virus & Sexual Health Service and School of Medicine, University of Brisbane, Brisbane, QLD, Australia; Rockhampton Blood Borne Virus & Sexual Health Service and School of Medicine, University of Brisbane, Brisbane, QLD, Australia; Department of Gastroenterology and Hepatology, Royal North Shore Hospital, St Leonards, NSW, Australia; Department of Gastroenterology and Hepatology, Royal North Shore Hospital, St Leonards, NSW, Australia; Department of Gastroenterology and Hepatology, Royal Hobart Hospital, Hobart, TAS, Australia; Bathurst Liver Clinic, Bathurst, NSW, Australia; Bathurst Liver Clinic, Bathurst, NSW, Australia; Cairns Sexual Health Service and James Cook University Cairns, St Cairns City, QLD, Australia; Department of Drug Health, Western Sydney Local Health District, Westmead, NSW, Australia; Department of Medicine, Cairns Hospital, Cairns, QLD, Australia; Centre for Infectious Diseases and Microbiology Laboratory Services, NSW Health Pathology-Institute of Clinical Pathology and Medical Research, Westmead Hospital, Westmead, NSW, Australia; Storr Liver Centre, The Westmead Institute for Medical Research, The University of Sydney and Westmead Hospital, Sydney, NSW, Australia; Storr Liver Centre, The Westmead Institute for Medical Research, The University of Sydney and Westmead Hospital, Sydney, NSW, Australia; Centre for Infectious Diseases and Microbiology, Sydney Infectious Diseases Institute, The University of Sydney at Westmead Hospital, Sydney, NSW, Australia

**Keywords:** antimicrobial resistance, antiviral therapy, direct acting antivirals, drug resistance, Hepatitis C

## Abstract

**Background:**

Hepatitis C virus (HCV) infection can now be cured with well-tolerated direct-acting antiviral (DAA) therapy. However, a potential barrier to HCV elimination is the emergence of resistance-associated substitutions (RASs) that reduce the efficacy of antiviral drugs, but real-world studies assessing the clinical impact of RASs are limited. Here, an analysis of the impact of RASs on retreatment outcomes for different salvage regimens in patients nationally who failed first-line DAA therapy is reported.

**Methods:**

We collected data from 363 Australian patients who failed first-line DAA therapy, including: age, sex, fibrosis stage, HCV genotype, NS3/NS5A/NS5B RASs, details of failed first-line regimen, subsequent salvage regimens, and treatment outcome.

**Results:**

Of 240 patients who were initially retreated as per protocol, 210 (87.5%) achieved sustained virologic response (SVR) and 30 (12.5%) relapsed or did not respond. The SVR rate for salvage regimens that included sofosbuvir/velpatasvir/voxilaprevir was 94.3% (n = 140), sofosbuvir/velpatasvir 75.0% (n = 52), elbasvir/grazoprevir 81.6% (n = 38), and glecaprevir/pibrentasvir 84.6% (n = 13). NS5A RASs were present in 71.0% (n = 210) of patients who achieved SVR and in 66.7% (n = 30) of patients who subsequently relapsed. NS3 RASs were detected in 20 patients (20%) in the SVR group and 1 patient in the relapse group. NS5B RASs were observed in only 3 patients. Cirrhosis was a predictor of relapse after retreatment, as was previous treatment with sofosbuvir/velpatasvir.

**Conclusions:**

In our cohort, the SVR rate for sofosbuvir/velpatasvir/voxilaprevir was higher than with other salvage regimens. The presence of NS5A, NS5B, or NS3 RASs did not appear to negatively influence retreatment outcomes.

Hepatitis C virus (HCV) infection is one of the leading causes of liver cirrhosis and hepatocellular carcinoma. The development of interferon-free direct-acting antiviral (DAA) therapy has revolutionized HCV treatment with sustained virologic response (SVR) rates of >95% and improved uptake because of oral therapy, shorter treatment duration, and fewer adverse effects [1]. In Australia, DAA therapy was listed on the Pharmaceutical Benefits Scheme in March 2016, with unrestricted access and community prescribing. By the end of 2022, more than 105 000 people had initiated DAA therapy, 56% of the total number of people who were infected in 2015 [2].

A potential barrier to meeting World Health Organization elimination targets for hepatitis C [3] is the emergence of resistance-associated substitutions (RASs) in the virus genome. The resulting change in amino acid sequence in viral proteins NS3, NS5A, or NS5B leads to reduced susceptibility to DAA therapy [4–6]. All modern DAA regimens include an NS5A inhibitor, so NS5A RASs hold the most clinical significance because they can reduce cure rates in previously untreated people with genotype 3 infection and/or cirrhosis [7]. Although the prevalence of RASs is low in untreated people, it increases in patients who fail initial DAA therapy because of selective pressure for antiviral resistance [6]. In clinical trials, the majority of people who fail first-line DAA treatment develop RASs in the virus [8, 9]. We have confirmed this previously in the real world, using data from Australia and the international SHARED consortium [10, 11]. In our large cohorts, although the background prevalence of NS5A RASs in untreated patients is approximately 30%, in patients who fail treatment this increases to 70%–80% [10–12]. This increase in NS5A RASs is of particular concern as they have minimal effect on viral fitness and so may persist for months to years after DAA failure [13], providing potential for transmission of drug-resistant viruses [14–16].

For those who do not respond to first-line therapy, until recently there were few options available for retreatment. The presence of RASs in NS5A impairs response to retreatment with most first-line DAA regimens, so patients who fail treatment with these drugs the first time and develop resistance are even more likely to fail again [5]. Early guidelines relied on treatment history to choose drugs with a different spectrum of activity, guided by virus resistance testing where available [17]. Retreatment outcomes have improved dramatically since the availability of sofosbuvir/velpatasvir/voxilaprevir, a pan-genotypic regimen that targets 3 HCV proteins (NS3, NS5A, and NS5B). In the POLARIS clinical trials in the United States, this salvage regimen achieved SVR rates >95% in patients with prior DAA treatment failure across all HCV genotypes [18, 19]. In Australia, sofosbuvir/velpatasvir/voxilaprevir was listed on the Pharmaceutical Benefits Scheme in April 2019 and has been another leap forward in hepatitis C treatment.

In line with most international guidelines [20, 21], current Australian guidelines recommend sofosbuvir/velpatasvir/voxilaprevir for retreating patients with prior DAA failure, or even multiple treatment failures [22]. However, before 2019, nonresponders to first-line therapy were prescribed various off-label salvage combinations of existing DAA regimens including: sofosbuvir/velpatasvir, glecaprevir/pibrentasvir, sofosbuvir + daclatasvir, or paritaprevir/ritonavir/ombitasvir + dasabuvir (PrOD), often in combination with ribavirin [17, 23]. Each of these treatments had the potential to select for further RASs in patients who failed, leading to a more resistant virus and further retreatment failure [4–6].

In 2020, we published the largest study to date (n = 572) on the prevalence of RASs in people failing treatment with modern DAA regimens containing an NS5A inhibitor [10]. Of note, 60% of the samples in that study were from people with HCV genotype 3, which is more difficult to treat and is relatively underrepresented in studies from the United States because of global variation in the genomic landscape of HCV [24]. In this study, we analyze the clinical effect of RASs on retreatment outcomes, using follow-up data for 363 patients in our cohort, 240 of whom received further antiviral treatment for their hepatitis C.

In Australia, several studies have examined baseline pretreatment HCV RASs [12, 25] but there are few data on RASs in patients who fail first-line treatment and their response to subsequent salvage treatments. This study spans the period before and after the introduction of sofosbuvir/velpatasvir/voxilaprevir in Australia, providing a rare opportunity to examine and compare real-world data of different salvage regimens on a nationwide scale.

## METHODS

Between 1 January 2017 and 30 June 2019, 572 people with detectable HCV RNA in their plasma, more than 12 weeks after completing DAA therapy, were referred to Westmead Hospital for HCV resistance testing, from more than 80 different treatment centers across Australia. Detailed protocols for HCV RNA extraction, amplification, and sequencing can be found in our previous report [10].

People in this cohort were followed up from June 2019 to June 2022. Clinical data were collected including age, sex, fibrosis stage, HCV genotype, details of failed first-line regimen, and subsequent salvage regimens. Retreatment outcomes were obtained by contacting treatment referral centers to obtain encrypted and deidentified data on retreatment, including salvage regimens used, completion date, and retreatment outcome (12-week SVR). A few patients were referred to Westmead Hospital again for repeat sequencing of HCV RASs.

Data analysis was performed using R, SankeyMATIC, and Microsoft Excel. Statistical analyses were performed using chi-square analysis as appropriate. *P* < .05 was considered statistically significant.

The protocol was approved by the Western Sydney Local Health District Human Research and Ethics Committee (LNR/17/WMEAD/484). This institutional ethics committee complies with the Declaration of Helsinki.

## RESULTS

### Retreatment Outcomes of Salvage Regimens

**Figure 1.**
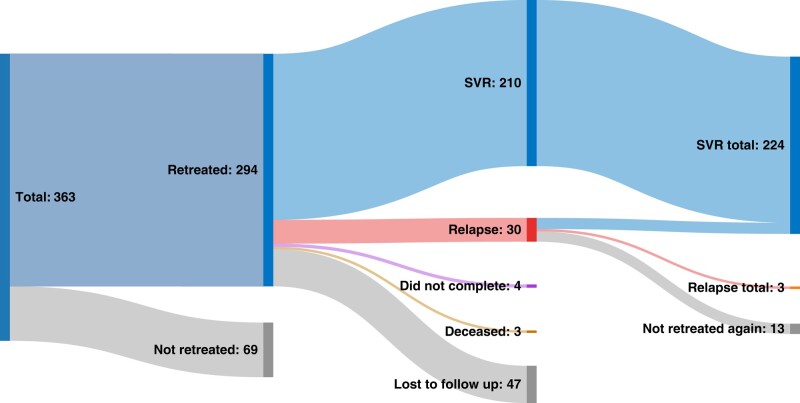
Retreatment outcomes of 294 patients who had failed initial DAA treatment (June 2022). Absolute numbers shown. Abbreviations: DAA, direct-acting antiretroviral; SVR, sustained virologic response.

Between June 2019 and June 2022, we followed and obtained data on clinical outcomes for 363 patients who had been referred to our center for HCV RNA sequencing after failing DAA therapy. Of these patients, 294 were retreated for their hepatitis C and 69 were not retreated. The current status of these retreated patients is shown in Figure 1. Of the 294 retreated patients, 240 (82%) completed retreatment, 4 (1.4%) did not complete initial retreatment, 3 (1.0%) were deceased, and 47 (16%) were lost to follow up.

Per protocol, 210/240 patients (87.5%) successfully achieved SVR with initial salvage therapy. Thirty patients (12.5%) relapsed or did not respond to initial salvage treatment. Of these 30, 17 patients (57%) received a second course of salvage treatment. Per protocol, 14/17 (82%) achieved SVR with the second retreatment, whereas 3 (18%) did not. By intention to treat, 210/294 patients (71.4%) achieved SVR with initial salvage therapy and 30/294 (10.2%) were confirmed relapse or nonresponders.

**Figure 2.**
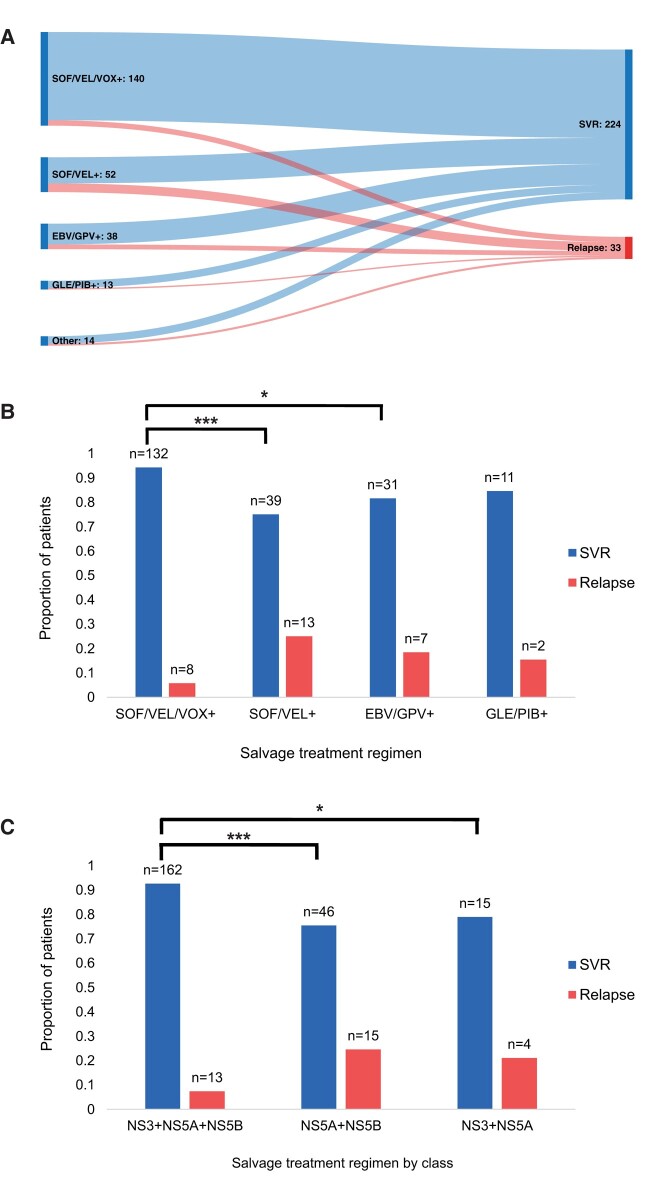
Response to various DAA salvage regimens in patients who failed first-line DAA therapy, represented as (*A*) a flow diagram and as (*B*) the proportion of patients who achieved SVR. *A*, Absolute numbers shown. Other regimens include: ledipasvir/sofosbuvir; ombitasvir/paritaprevir/ritonavir + dasabuvir + sofosbuvir + ribavirin; sofosbuvir + daclatasvir + ribavirin; sofosbuvir + ribavirin. *B*, Statistical significance for retreatment outcome reported, *P* ≤ .05 (*), *P* ≤ .001 (***). Abbreviations: DAA, direct-acting antiretroviral; EBV, elbasvir; GLE, glecaprevir; GPV, grazoprevir; PIB, pibrentasvir; SOF, sofosbuvir; SVR, sustained virologic response; VEL, velpatasvir; VOX, voxilaprevir. + denotes with or without addition of ribavirin or another DAA. *C*, Response to various salvage regimens grouped by DAA class. Represented as the proportion of patients who achieved SVR, with absolute numbers shown. Statistical significance for retreatment outcome reported, *P ≤ .05, ***P ≤ .001.

The use of and response to various DAA salvage regimens is summarized in Figure 2*A*, with the proportion of patients who achieved SVR or relapsed with each regimen shown in Figure 2*B* and a comparison by DAA class in Figure 2*C*. Further details are available in Table 1.

**Table 1.** Detailed Summary of Various DAA Salvage Regimens Used for Patients who Failed First-line DAA Therapy

Salvage Regimen	SVR	Relapse	Total	SVR Rate (%)
Sofosbuvir/velpatasvir/voxilaprevir	125	6	131	95.42
Sofosbuvir/velpatasvir/voxilaprevir + ribavirin	7	2	9	77.78
Sofosbuvir/velpatasvir	28	7	35	80.00
Sofosbuvir/velpatasvir + ribavirin	11	6	17	64.71
Elbasvir/grazoprevir	6	1	7	85.71
Elbasvir/grazoprevir + ledipasvir/sofosbuvir	0	1	1	…
Elbasvir/grazoprevir + sofosbuvir	6	3	9	66.67
Elbasvir/grazoprevir + sofosbuvir + ribavirin	19	1	20	95.00*
Elbasvir/grazoprevir + ribavirin	0	1	1	…
Glecaprevir/pibrentasvir	7	2	9	77.78
Glecaprevir/pibrentasvir + sofosbuvir	1	0	1	…
Glecaprevir/pibrentasvir + sofosbuvir + ribavirin	3	0	3	100
Ledipasvir/sofosbuvir	5	1	6	83.33
Ombitasvir/paritaprevir/ritonavir + dasabuvir	1	0	1	…
Ombitasvir/paritaprevir/ritonavir + dasabuvir + sofosbuvir + ribavirin	1	0	1	…
Ombitasvir/paritaprevir/ritonavir + dasabuvir + ribavirin	1	0	1	…
Sofosbuvir/daclatasvir	0	1	1	…
Sofosbuvir/daclatasvir + ribavirin	2	0	2	100
Sofosbuvir + ribavirin	1	1	2	50.00

Absolute numbers shown. SVR rate was calculated for regimens where n > 1. Statistical significance for retreatment outcome when adding ribavirin reported, *P ≤ .001.

Abbreviations: DAA, direct-acting antiretroviral; SVR, sustained virologic response.

The most prevalent salvage regimen was sofosbuvir/velpatasvir/voxilaprevir ± ribavirin, with 140 patients retreated with this regimen and 132/140 achieving SVR (94.3%). With sofosbuvir/velpatasvir ± ribavirin, 39/52 (75.0%) achieved SVR. With elbasvir/grazoprevir-based regimens, 31/38 (81.6%) achieved SVR. With glecaprevir/pibrentasvir-based regimens, 11/13 (84.6%) achieved SVR.

Retreatment with sofosbuvir/velpatasvir/voxilaprevir ± ribavirin was more likely to achieve SVR compared to sofosbuvir/velpatasvir-based regimens, χ^2^ (1, N = 192) = 14.4773, *P* = .000142; elbasvir/grazoprevir-based regimens, χ^2^ (1, N = 178) = 6.2535, *P* = .012395; or all other regimens combined, χ^2^ (1, N = 243) = 13.42, *P* = .0002. There was no statistical significance when comparing between other salvage regimens.

When comparing outcomes by DAA class, triple therapy with NS3 + NS5A + NS5B inhibitors was far more effective in achieving SVR compared to dual therapy with either NS5A + NS5B inhibitors, χ^2^ (1, N = 236) = 12.74, *P* = .00036; or NS3 + NS5A inhibitors, χ^2^ (1, N = 194) = 3.98, *P* = .046. There was no statistically significant difference between NS5A + NS5B and NS3 + NS5A regimens. For this analysis, the combination of PrOD was grouped with NS3 + NS5A inhibitors because dasabuvir is a nonnucleos(t)ide NS5B inhibitor with a low barrier to resistance. When PrOD was combined with sofosbuvir as a salvage therapy, it was grouped with NS3 + NS5A + NS5B.

### The Impact of Adding Ribavirin on Retreatment Outcomes

Overall, the addition of ribavirin did not increase the SVR rate, but this varied according to DAA regimen. Adding ribavirin to elbasvir/grazoprevir + sofosbuvir increased the SVR rate from 66.7% to 95% (*P* = .04). In contrast, adding ribavirin to either sofosbuvir/velpatasvir or sofosbuvir/velpatasvir/voxilaprevir did not increase SVR rates, which were in fact lower in the groups treated with ribavirin. This is most likely because of the higher rates of cirrhosis in patients prescribed ribavirin with these DAAs. A total of 79% of patients receiving sofosbuvir/velpatasvir + ribavirin had cirrhosis, compared with 36% in the group not prescribed ribavirin, χ^2^ (1, N = 55) = 19.98, *P* = <.001. A similar trend was seen in patients receiving sofosbuvir/velpatasvir/voxilaprevir; 50% of patients prescribed ribavirin had cirrhosis compared with 36% of those not prescribed ribavirin (*P* = .48, nonsignificant). There was no trend toward increased cirrhosis in patients prescribed elbasvir/grazoprevir + sofosbuvir + ribavirin compared with those prescribed elbasvir/grazoprevir + sofosbuvir.

### Sofosbuvir/Velpatasvir/Voxilaprevir is Less Effective in Patients Who Have Previously Failed Sofosbuvir/Velpatasvir

Although SVR rates were excellent for sofosbuvir/velpatasvir/voxilaprevir overall (95%), there was an impact of previous treatment regimen. In patients who had previously failed treatment with sofosbuvir/velpatasvir, the SVR rate was 75% compared with 95% for other previous regimens, χ^2^ (1, N = 116) = 8.49, *P* = <.01.

### The Impact of HCV Genotype on Retreatment Outcomes

**Figure 3.**
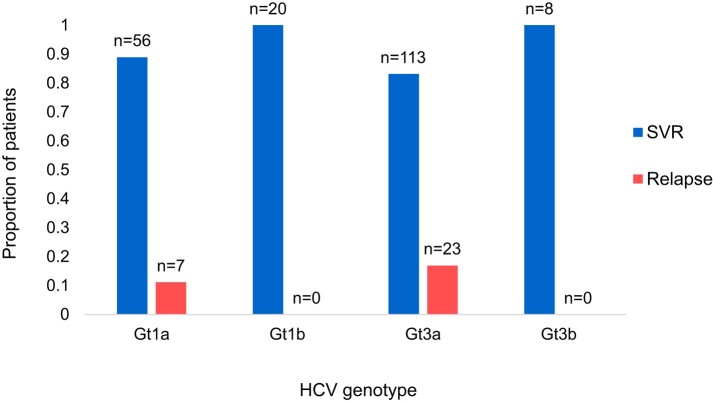
Proportion of patients who achieved SVR or relapsed with DAA retreatment for each HCV isolate genotype. Abbreviations: DAA, direct-acting antiretroviral; Gt, genotype, HCV, hepatitis C virus; SVR, sustained virologic response.

A comparison of retreatment outcomes between HCV genotypes 1 and 3 is shown in Figure 3. A total of 56/63 patients (88.9%) with HCV genotype 1a infection achieved SVR, whereas all 20 patients (100%) with genotype 1b achieved SVR. A total of 113/136 patients (83.1%) with genotype 3a and all 8/8 patients (100%) with genotype 3b achieved SVR. Although there was a trend toward worse retreatment outcomes for people with genotype 3a infection, there was no statistically significant difference between HCV genotypes.

### The Impact of Liver Fibrosis on Retreatment Outcomes

**Figure 4.**
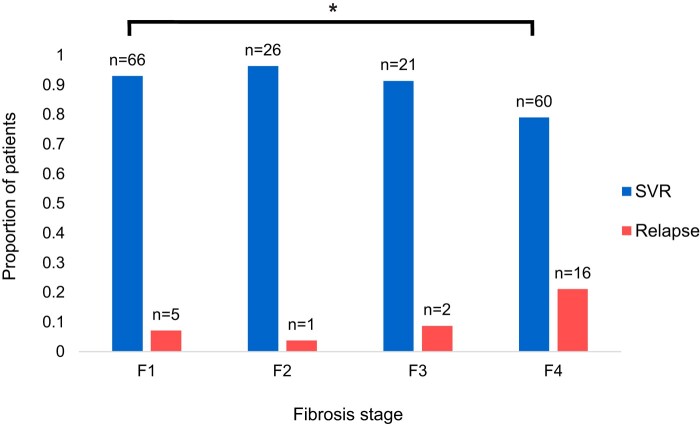
Proportion of patients who achieved SVR or relapsed with DAA retreatment for each fibrosis stage. Statistical significance for retreatment outcome reported, *P ≤ .05 (). Abbreviations: DAA, direct-acting antiretroviral; SVR, sustained virologic response.

Retreatment outcomes for each liver fibrosis stage in patients with reported fibrosis scores are shown in Figure 4. Fibrosis scores were available for 197 patients based on transient elastography or liver biopsy. For patients with F1 liver fibrosis, 66/71 (93.0%) achieved SVR with DAA salvage therapy. Twenty-six of 27 (96.3%) patients with F2 and 21/23 (91.3%) with F3 achieved SVR. In patients with liver cirrhosis (F4), 60/76 achieved SVR (79.0%). There was a negative and statistically significant effect of cirrhosis (F4 vs F1) on retreatment outcomes, χ^2^ (1, N = 147) = 5.8844, *P* = .015276. Comparisons between other fibrosis stages were not statistically significant by chi-square with Yates correction.

### The Impact of NS5A RASs on Retreatment Outcomes

**Figure 5.**
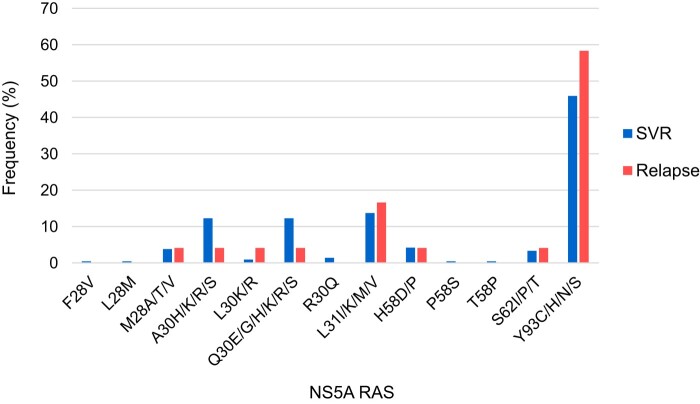
Frequency of NS5A RASs in patients who achieved SVR or relapsed with DAA retreatment. Frequency presented as a proportion of total NS5A RASs detected in the SVR group (n = 149) and relapse group (n = 20). Abbreviations: DAA, direct-acting antiretroviral; RAS, resistance-associated substitution; SVR, sustained virologic response.

For all patients, initial sequencing for HCV RASs was completed after failing first-line DAA treatment before retreatment with salvage regimens. NS5A RASs were detected in 149/210 (71.0%) patients who went on to achieve SVR and in 20/30 (66.7%) patients who subsequently relapsed. In the SVR group, 104 patients had an NS5A RAS at a single site, 37 had RASs at 2 sites, and 8 had RASs at 3 or more sites. In the relapse group, 16 had a RAS at a single site and 4 had RASs at 2 sites. A breakdown of the frequency of detected NS5A RASs is available in Figure 5. RASs at residue Y93 remained the most common RAS in both groups.

The presence of NS5A RASs reduced SVR rates for patients treated with sofosbuvir/velpatasvir ± ribavirin. The SVR rate was 58.3% in those with NS5A RASs, compared with 85.7% in those without, χ^2^ (1, *N* = 52) = 4.924, *P* = <.05.

There was no effect of NS5A RASs on SVR rates for sofosbuvir/velpatasvir/voxilaprevir ± ribavirin, or elbasvir/grazoprevir + sofosbuvir ± ribavirin. It was not possible to look for an effect with other retreatment regimens because of small patient numbers.

There was no single RAS that predicted worse retreatment outcomes. The presence of more than 1 RAS also did not predict worse retreatment outcomes.

### The Impact of NS3 and NS5B RASs on Retreatment Outcomes

NS3 RASs were detected in 20/210 (9.5%) of patients in the SVR group. These NS3 RASs include substitutions at residues V55, Y56, Q80, S122, R155, A156, D168, Q168, and I170. NS3 RASs were detected in 1 patient in the relapse group (T54S, A155K).

NS5B RASs were detected in 2 patients who went on to achieve SVR. These RASs included S282T and S556G. NS5B RASs (G558E, D559N) were detected in 1 patient who relapsed.

Although patient numbers were small, there was no apparent impact of NS3 or NS5B RASs on retreatment outcomes.

## DISCUSSION

To achieve global elimination of hepatitis C, we need to understand the barriers to treatment success. One potential barrier is antiviral drug resistance, conferred by RASs in the HCV genome. This is particularly a problem for people who fail first-line DAA therapy because we and others have shown high rates of RASs after DAA failure can hinder subsequent retreatment [8–12]. Clinical trials have shown high cure rates in people retreated with sofosbuvir/velpatasvir/voxilaprevir [18, 19], but this combination is not available in all countries and there are relatively few data on retreatment outcomes in the real world. We previously published the largest real-world study of RAS prevalence among people failing a modern, interferon-free regimen DAA regimen [10]. This cohort of 572 people represented approximately 20% of all those in Australia who failed DAA therapy between January 2017 and June 2019. In this follow-up study of 363 people (63% of the initial cohort), 294 of whom (81%) were retreated for their hepatitis C, we compared different DAA salvage regimens because we captured a unique window spanning the period before and after the availability of sofosbuvir/velpatasvir/voxilaprevir for salvage therapy. Our cohort also contains a high proportion of people with genotype 3 infection, who are more difficult to treat with current DAAs and are typically underrepresented in other studies.

Among the 240 people for whom retreatment outcome data were available, 131 patients (55%) were treated with sofosbuvir/velpatasvir/voxilaprevir, with an SVR rate of 95% across HCV genotypes 1, 2, 3, 4, and 6. This was slightly lower than the 96%–98% SVR rate in the phase 3 POLARIS-1 and POLARIS-4 clinical trials of sofosbuvir/velpatasvir/voxilaprevir [18, 19], but similar to other large real-world salvage studies from the United Kingdom (SVR 90%, n = 144), USA (91%, n = 551), Spain (95%, n = 137), and Northern Italy (SVR 96%, n = 179) [26–29].

The SVR rate among patients treated with sofosbuvir/velpatasvir/voxilaprevir (95%) was higher than for patients retreated with older DAA combinations. We confirmed that DAA combinations with 3 drug classes (NS3, NS5A, and NS5B inhibitors) gave higher SVR rates than 2-class combinations. The SVR rate was 75% for sofosbuvir/velpatasvir ± ribavirin and 82% for elbasvir/grazoprevir-based regimens, although there was a large variation in the elbasvir/grazoprevir group (66.7%–95.0%), depending on which other DAAs were added. The most common combination was the 3-class combination of elbasvir (NS5A inhibitor)/grazoprevir (NS3 inhibitor) plus sofosbuvir (NS5B inhibitor); adding ribavirin to this combination increased SVR rates from 67% (6/9) to 95% (19/20, *P* < .05), consistent with a previous study, which achieved SVR in 96% of patients receiving this combination [30].

The main predictor of retreatment failure was liver cirrhosis, with an SVR rate of 79% compared with 93% for those with F1 fibrosis (*P* < .05). Cirrhosis is a major prognostic factor for DAA treatment outcomes [31] and reduced SVR rates (81%–89%) even with sofosbuvir/velpatasvir/voxilaprevir retreatment [26–29]. The relatively low prevalence of cirrhosis in our cohort (30%) explains the higher overall SVR rate (95%) with sofosbuvir/velpatasvir/voxilaprevir retreatment, compared with the 85% SVR reported in Australian patients enrolled in the sofosbuvir/velpatasvir/voxilaprevir compassionate access scheme, 78% of whom had cirrhosis [32].

Despite the high prevalence of genotype 3 infection (60%), we did not observe a significant impact of HCV genotype on retreatment outcomes. Previous studies have shown reduced SVR rates in patients with genotype 3 [26, 29], or genotype 1a/genotype 3 infection [27], compared with other genotypes. Consistent with this, a Taiwanese study of sofosbuvir/velpatasvir/voxilaprevir retreatment in people with mostly genotype 1b or genotype 2 infections reported a very high SVR rate of 97% [33]. We observed a trend toward reduced SVR with genotype 3a and to a lesser degree genotype 1a, but these did not reach statistical significance, as was the case in the US study that found SVR rates of 90.7% (429/473) for genotype 1 and 91.3% (42/46) for genotype 3 [28].

We observed no benefit of adding ribavirin to sofosbuvir/velpatasvir/voxilaprevir. This combination was used in only 9 patients, 2 of whom failed retreatment (SVR 78%). All 9 patients had genotype 3a infection and both retreatment failures had liver cirrhosis, likely explaining the trend toward reduced SVR rate, although the small sample size limits definite conclusions.

The presence of RASs impacted retreatment with some 2-class DAA combinations, but not for 3-class combinations (containing NS3, NS5A, and NS5B inhibitors). For patients retreated with sofosbuvir/velpatasvir, NS5A RASs reduced SVR rates from 86% to 58.3%, but they had no impact on elbasvir/grazoprevir + sofosbuvir or sofosbuvir/velpatasvir/voxilaprevir. Overall, there was a trend toward more frequent NS5A RASs in patients who went on to achieve SVR (71.0%) than in those who relapsed (66.7%); no particular RAS pattern influenced retreatment success. NS3 RASs were only observed in 21 patients, 20 of whom achieved SVR and 1 who relapsed. NS5B RASs were detected in only 3 patients and did not correlate with outcomes. Our findings are consistent with previous reports that the presence of RASs had no impact on retreatment with sofosbuvir/velpatasvir/voxilaprevir [29, 34, 35], with no consistent RAS pattern in treatment failures [36].

Importantly, we observed reduced response to retreatment with sofosbuvir/velpatasvir/voxilaprevir in patients who had previously failed sofosbuvir/velpatasvir (SVR 75% compared with 95% for other regimens). This raises concerns for retreating patients exposed to this common first-line regimen, particularly those with genotype 3a infection and cirrhosis. One promising approach to retreat patients who fail sofosbuvir/velpatasvir/voxilaprevir is the combination of glecaprevir/pibrentasvir + sofosbuvir ± ribavirin. This combination has greater in vitro activity against virus containing the high-level Y93H RAS in NS5A and has shown promising results in a small number of patients [36, 37], although these need to be confirmed in larger clinical trials.

In summary, the 3-class combination of sofosbuvir/velpatasvir/voxilaprevir was superior for retreating DAA-experienced patients compared with other salvage regimens. The addition of ribavirin did not improve treatment outcomes, although it was beneficial in patients treated with older combinations based on elbasvir/grazoprevir. This is important because it provides an alternative salvage regimen in settings where sofosbuvir/velpatasvir/voxilaprevir is not available. Liver cirrhosis was a negative prognostic factor for retreatment outcomes, as was prior treatment with sofosbuvir/velpatasvir. Although RASs in NS5A impacted retreatment with some DAA combinations, they appear to have minimal impact on salvage therapy with sofosbuvir/velpatasvir/voxilaprevir. Nonetheless it is important to monitor for increasing prevalence and impact of RASs in the community, particularly among people in high-risk settings who fail multiple courses of DAA therapy and could potentially transmit drug-resistant viruses to others. Genotype 3a is a particular concern because of its reduced susceptibility to DAA therapy, the high prevalence of NS5A RASs, and a recent report of sofosbuvir resistance (S282T RAS in NS5B) circulating in Pakistan [38]. Indeed, it is possible to induce replication competent, multidrug resistant virus in vitro [39], reinforcing the need for ongoing vigilance to maintain the efficacy of current treatments as we work toward global elimination of hepatitis C.

***Patient consent statement*.** The protocol was approved by the Western Sydney Local Health District Human Research and Ethics Committee (LNR/17/WMEAD/484). This institutional ethics committee complies with the Declaration of Helsinki. The study does not include factors necessitating patient consent as only deidentified data were collected from existing health records, with no direct patient contact.

***Financial support*.** This work received funding from the following: the National Health and Medical Research Council Ideas Grant APP2002565 and Program Grant APP1149976 (JG); Australian Centre for HIV and Hepatitis Virology Research and the Robert W. Storr Bequest to the Sydney Medical Foundation, The University of Sydney.

All authors have submitted the ICMJE Form for Disclosure of Potential Conflicts of Interest. Conflicts that the editors consider relevant to the content of the manuscript have been disclosed.
